# SerpinB10, a Serine Protease Inhibitor, Is Implicated in UV-Induced Cellular Response

**DOI:** 10.3390/ijms22168500

**Published:** 2021-08-07

**Authors:** Hajnalka Majoros, Barbara N. Borsos, Zsuzsanna Ujfaludi, Zoltán G. Páhi, Mónika Mórocz, Lajos Haracska, Imre Miklós Boros, Tibor Pankotai

**Affiliations:** 1 Institute of Pathology, Faculty of Medicine, University of Szeged, 1 Állomás utca, H-6725 Szeged, Hungary; majoros.hajnalka@szte.hu (H.M.); borsos.barbara.nikolett@szte.hu (B.N.B.); odianya@bio.u-szeged.hu (Z.U.); pahi.zoltan@med.u-szeged.hu (Z.G.P.); 2 HCEMM-BRC Mutagenesis and Carcinogenesis Research Group, Institute of Genetics, Biological Research Centre, H-6726 Szeged, Hungary; morocz@brc.hu (M.M.); haracska.lajos@brc.hu (L.H.); 3 Department of Biochemistry and Molecular Biology, Faculty of Science and Informatics, University of Szeged, H-6726 Szeged, Hungary; borosi@bio.u-szeged.hu; 4 Institute of Biochemistry, Biological Research Centre, H-6726 Szeged, Hungary

**Keywords:** SerpinB10, Bomapin, serine protease inhibitor, replication, replication stress, UV damage

## Abstract

UV-induced DNA damage response and repair are extensively studied processes, as any malfunction in these pathways contributes to the activation of tumorigenesis. Although several proteins involved in these cellular mechanisms have been described, the entire repair cascade has remained unexplored. To identify new players in UV-induced repair, we performed a microarray screen, in which we found *SerpinB10* (*SPB10*, *Bomapin*) as one of the most dramatically upregulated genes following UV irradiation. Here, we demonstrated that an increased mRNA level of *SPB10* is a general cellular response following UV irradiation regardless of the cell type. We showed that although SPB10 is implicated in the UV-induced cellular response, it has no indispensable function in cell survival upon UV irradiation. Nonetheless, we revealed that SPB10 might be involved in delaying the duration of DNA repair in interphase and also in S-phase cells. Additionally, we also highlighted the interaction between SPB10 and H3. Based on our results, it seems that SPB10 protein is implicated in UV-induced stress as a “quality control protein”, presumably by slowing down the repair process.

## 1. Introduction

Our genome is constantly exposed to endogenous and exogenous sources of damage. One of them is UV radiation, which has many harmful physiological and biological consequences, such as premature aging, immunosuppression, overactivation of inflammatory processes, DNA damage, and even the activation of apoptosis [1,2]. Understanding the mechanism of UV-induced DNA damage and the subsequent cellular response is indispensable, as a malfunction in the DNA repair process often leads to tumorigeneses [1,2]. UV irradiation can induce the formation of 8-hydroxyguanine, DNA-protein crosslinks, or abasic sites or lead to the appearance of cyclobutane-pyrimidine dimers (CPDs) or 6–4 pirimidine-pirimidon photoproducts (6–4 PPs) [3,4,5,6]. The altered DNA structures cause problems during DNA replication, since the replisome quickly and accurately copies billions of DNA bases in each cell cycle, including the damaged bases. Furthermore, altered DNA structures can lead to the formation of mismatches, particularly when the template DNA has been damaged. Therefore, the effectiveness of DNA repair is crucial before the replisome can bypass the damaged nucleotides. To rescue the stalled replication machinery, eukaryotic cells have evolved error-prone translesion (TLS) DNA polymerases, which can pass through the lesions. However, to keep these low-fidelity TLS polymerases away from the undamaged DNA, interaction is required between them and the proliferating cell nuclear antigen (PCNA), which serves as a binding platform for proteins involved in the recognition and repair of damage [7,8,9,10,11,12,13]. One of the main steps in the exchange of replicative polymerases to TLS DNA polymerases at stalled replication forks is mono-ubiquitylation; hence, the activation of PCNA, mediated by Rad6 and Rad18 ubiquitin ligases, is a crucial step for successful repair [7,10,14,15,16,17]. Any malfunction in these processes can lead to the accumulation of DNA mutations or chromosomal rearrangements or can affect chromosome segregation, resulting in tumorous malformations [18].

Our results and recent studies also showed that UV irradiation induces robust gene expression changes in the Hker E6SFM keratinocyte cell line [19,20,21,22]. Among the upregulated genes, we identified several members of the B clade of the Serpin superfamily, including *SerpinB2*, *SerpinB10*, and *SerpinB13* [19]. The members of the SerpinB (SPB) family are mainly intracellular proteins, and it has been demonstrated that they can regulate various processes, such as inflammation, immune function, mucous production, apoptosis, tumor metastasis, and autoimmunity [23,24,25,26,27]. Nonetheless, only a limited amount of data are available concerning their regulatory role in DNA repair [19,20,28,29,30].

In the last decades, several studies have described proteins with dual, contradictory functions in tumor formation and metastasis progression. Several proteins have been shown to facilitate tumor formation according to some cancer types, and to inhibit it in other tumor classes. SerpinB10 (SPB10), also known as Bomapin, is a redox-sensitive nucleocytoplasmic protein that promotes proliferation of hematopoietic and myeloid leukemia cells under basal conditions, although it also enhances apoptosis following withdrawal of growth factors [31]. The dual function of Bomapin has also been described in several cancer types, such as lung cancer, in which *SPB10* mRNA expression is increased, and breast cancer, in which it is decreased compared with healthy cells [32]. In addition, it has been also described that missense mutation of *Bomapin* can lead to the formation of prostate cancer [33]. 

In this study, we demonstrated that both the mRNA and protein levels of SPB10 are increased as a response to UV radiation, suggesting the role of SPB10 in UV-induced DNA damage repair. From our results, we presume that SPB10 is involved in the mediation of DNA repair upon UV radiation in interphase and also in S-phase cells. Additionally, SPB10 interaction with H3 histone can also be detected, suggesting its chromatin association.

## 2. Results

### 2.1. Expression of Several Members of the SerpinB Superfamily, Including SPB10, Is Increased Following UV Irradiation

Environmental stress factors frequently induce DNA damage, which initiates the activation of DNA repair. These processes are tightly regulated, and post-translational regulation of the participating proteins is required for their proper function. Recently, we demonstrated by microarray experiment that UV irradiation contributes to the overexpression of certain members of the *SerpinB* family, including *SerpinB2* (*SPB2*), *SerpinB10* (*SPB10*), and *SerpinB13* (*SPB13*), in Hker E6SFM cells, with 1.495-, 4.253-, and 1.180-fold increase (log_2_) upon UV irradiation, respectively (Figure S1).

**Figure 1:**
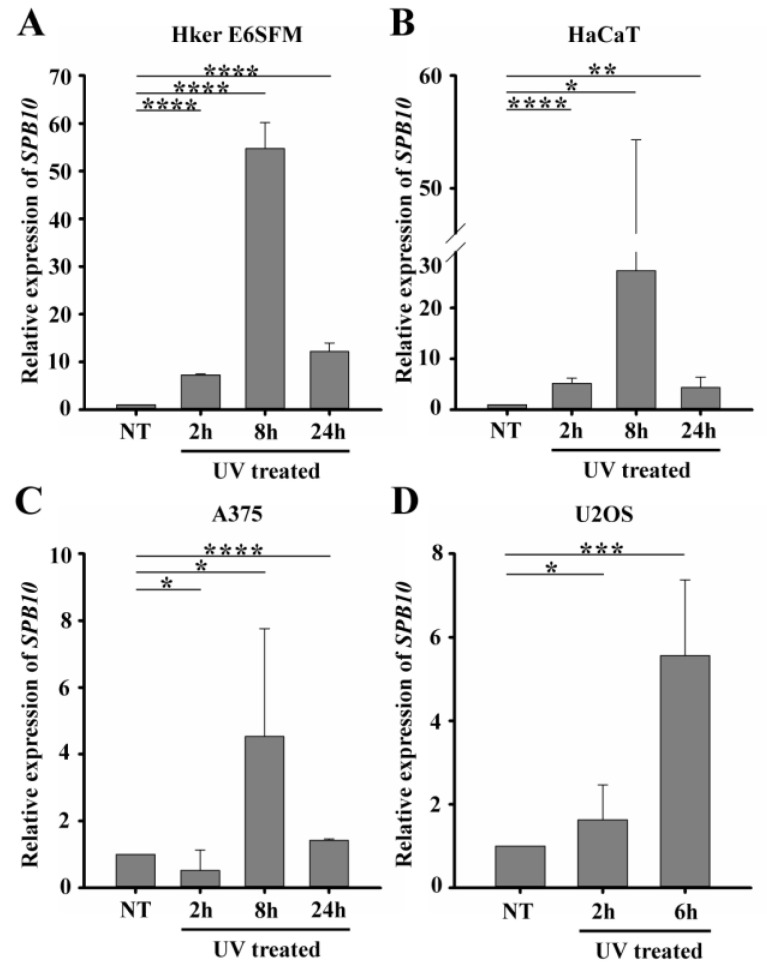
UV irradiation triggers elevation of *SPB10* mRNA: Relative expression levels of *SPB10* gene in (**A**) Hker E6SFM keratinocyte, (**B**) HaCaT keratinocyte, (**C**) A375 melanoma, and (**D**) U2OS osteosarcoma cells analyzed by qPCR. Data were normalized to 18S RNA and compared to control (NT). Means and standard deviations of three independent experimental triplicates are indicated as fold changes. Asterisks indicate statistical significance between datasets (*t*-test, * *p* ≤ 0.05, ** *p* ≤ 0.01, *** *p* ≤ 0.001, **** *p* ≤ 0.0001).

To verify the microarray data, we first performed quantitative PCR (qPCR) on Hker E6SFM cells to measure the mRNA level of *SPB10* in basal conditions and upon UV irradiation. We observed an increased expression of *SPB10* 2 and 8 h after UV irradiation compared with the control. The elevated mRNA level decreased 24 h after the UV radiation; however, it remained 12.2 times higher compared with the control (Figure 1A).

To determine whether elevated gene expression of *SPB10* could be observed in other cell lines as well, we performed qPCR experiments following UV irradiation for 2, 8, and 24 h and with untreated samples in HaCaT keratinocyte and A375 melanoma cells. Similar to Hker E6SFM, we detected elevated *SPB10* mRNA levels after UV irradiation in both cell lines (Figure 1B,C).

In order to reveal whether SPB10 plays a general role following UV radiation, we involved the U2OS osteosarcoma cell line in our study and performed the above-described experiment, since U2OS is generally used to examine DNA damage-induced cellular responses. We detected elevated *SPB10* mRNA levels 2 and especially 6 h after irradiation compared with the control samples in this cell line (Figure 1D). All of these results might indicate that the overexpression of *SPB10* upon UV irradiation is not cell-line-specific, but a general cellular response.

### 2.2. SPB10 Is Dispensable for Cell Survival but Influences DNA Repair Kinetics in Interphase Cells upon UV Irradiation

**Figure 2:**
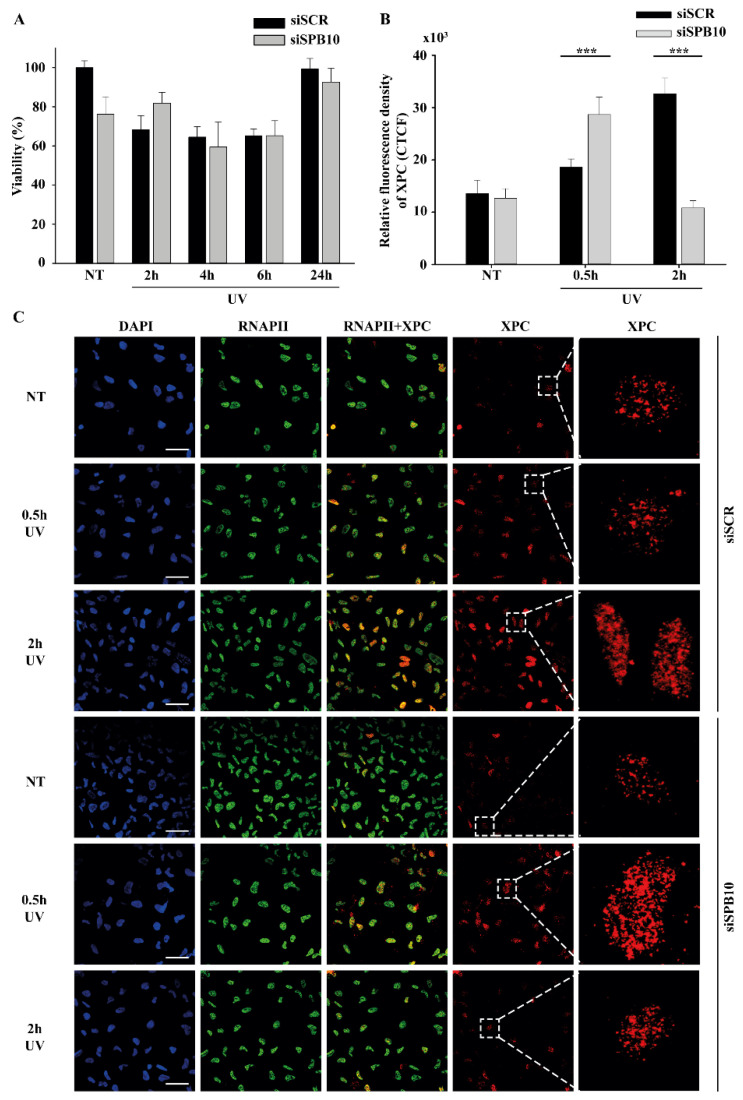
SPB10 is dispensable for cell survival but influences DNA repair kinetics in interphase cells upon UV irradiation: (**A**) Diagram represents cell viability (%) upon UV irradiation (2, 4, 6, and 24 h) of non-targeting (siSCR) and *SPB10* siRNA-silenced U2OS cells. (**B**) Quantification of relative fluorescence intensity (CTCF) of Xeroderma Pigmentosum C (XPC) protein detected on non-targeting (siSCR) and *SPB10* siRNA-silenced interphase U2OS cells in basal conditions (NT) and following UV irradiation (0.5 and 2 h). Asterisks indicate statistical significance between datasets (Mann–Whitney test, *** *p* ≤ 0.001). (**C**) Representative images of XPC protein (red) binding presumably to Nucleotide Excision Repair (NER) foci in non-targeting (siSCR, upstream) and *SPB10* siRNA-silenced (*siSPB10*, downstream) U2OS cells in basal conditions (NT) and after UV irradiation (0.5 and 2 h). Only the chromatin-bound proteins were visualized by CSK-immunocytochemistry. DAPI (blue) was used to visualize the nuclei, and RNA Polymerase II (RNAPII) (green) was used as control. Scale bars represent 200 µm. For each condition, a higher magnification of a single cell is shown on the right side of the figure.

Since our qPCR data suggested that SPB10 plays a role in UV-induced cellular responses, we examined whether it has any effect on cell survival in response to UV irradiation. To examine this hypothesis, we performed a viability assay on non-targeting scrambled siRNA (siSCR) and *SPB10* siRNA-silenced U2OS cells and determined the ratio of live and dead cells 2, 4, 6, and 24 h after UV radiation using Trypan blue staining (Figure 2A). As expected, we counted a reduced number of cells immediately after UV irradiation. Nonetheless, at later time points (24 h after exposure), the amount of living cells increased, as by that point, the cells had been already recovered. However, we did not find any significant differences between the numbers of non-targeting (siSCR) and *SPB10* siRNA-silenced cells, which indicates that SPB10 might have no effect on cell survival following UV irradiation.

Although we did not find any changes in cell survival, showing that the SPB10 is not crucial for the UV-induced repair, it can still act as a fine-tune regulator protein in Nucleotide Excision Repair (NER). To test this, we monitored the repair kinetics of NER upon UV irradiation, by following the binding of the Xeroderma Pigmentosum C (XPC) protein presumably to the site of DNA damage by using CSK-immunocytochemistry of non-targeting (siSCR) and *SPB10* siRNA-silenced U2OS cells. As we expected, the number of XPC foci was elevated after 30 min and 2 h UV irradiation compared with the control sample in non-targeting (siSCR) U2OS cells. The repair kinetics were shifted in *SPB10* siRNA-silenced cells 30 min following UV irradiation, while they were decreased 2 h post-UV compared with control (Figure 2B,C). Based on these results, we assumed that SPB10 could influence the XPC binding, and the loss of SPB10 accelerates the repair of UV-induced damage. 

### 2.3. SPB10 Influences DNA Repair Kinetics in S-Phase Cells upon UV Irradiation

Our data suggest that SPB10 may take part in regulating the repair of UV-induced DNA damage. UV-related T-T dimer formation can also result in replication block, which can be resolved by translesion (TLS) DNA polymerases recruited by the proliferating cell nuclear antigen (PCNA) protein [18,34]. 

To reveal whether SPB10 is involved in this process, we first performed co-immunoprecipitation (co-IP) to study the association between SPB10 and PCNA. However, we did not detect interaction between these two proteins (data not shown).

**Figure 3:**
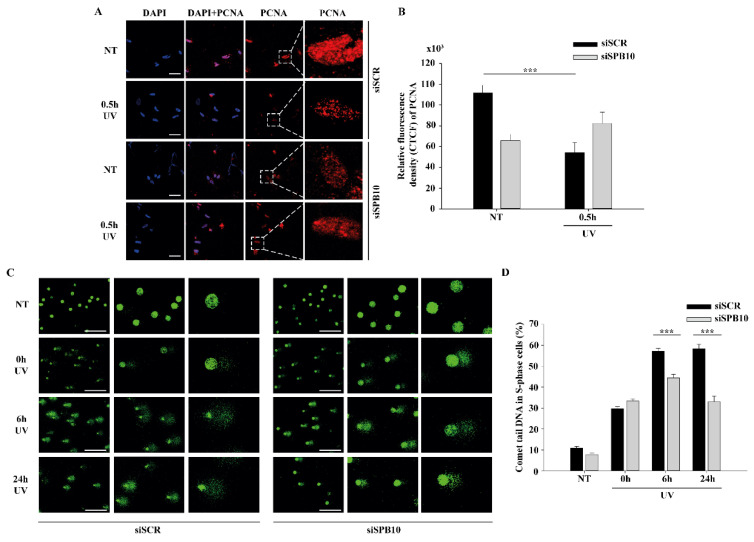
SPB10 influences DNA repair kinetics in S-phase cells upon UV irradiation: (**A**) Representative images of PCNA protein (red) binding in non-targeting (siSCR, upstream) and *SPB10* siRNA-silenced (*siSPB10*, downstream) U2OS cells in basal conditions (NT) and after UV irradiation (0.5 h). Only the chromatin-bound proteins were visualized by CSK-immunocytochemistry. DAPI (blue) was used to visualize the nuclei. Scale bars represent 200 µm. For each condition, a higher magnification of a single cell is shown on the right side of the figure. (**B**) Quantification of relative fluorescence intensity (CTCF) based on measured foci number of proliferating cell nuclear antigen (PCNA) protein detected of non-targeting (siSCR) and *SPB10* siRNA-silenced U2OS cells in basal conditions (NT) and following UV irradiation (0.5 h). Asterisks indicate statistical significance between datasets (Mann–Whitney test, *** *p* ≤ 0.001). (**C**) Representative images of comet tail formation in non-targeting (siSCR, left) and *SPB10* siRNA-silenced (*siSPB10*, right) bromodeoxyuridine (BrdU)-labelled S-phase U2OS cells in basal conditions (NT) and after UV irradiation (0, 6, and 24 h). Scale bars represent 200 µm. (**D**) Quantification of comet DNA tails detected on non-targeting (siSCR) and *SPB10* siRNA-silenced S-phase U2OS cells in basal conditions and following UV irradiation (0, 6, and 24 h). Asterisks indicate statistical significance between datasets (Mann-Whitney test, *** *p* ≤ 0.001).

Next, we tested whether SPB10 has any influence on the chromatin-bound PCNA recruitment upon UV irradiation. We created CSK-immunocytochemistry of non-targeting (siSCR) and *SPB10* siRNA-silenced U2OS cells and we monitored the level of chromatin-bound PCNA in siSCR cells and 30 min after UV treatment. We observed less chromatin-bound PCNA upon UV irradiation in non-targeting (siSCR) siRNA treated U2OS cells, which might be caused by replication fork collapse mechanism. However, in the absence of SPB10, we could not observe this reduction in the PCNA level upon UV radiation (Figure 3A,B). According to these results, we hypothesized that SPB10 might involve in the fine-tuning of the UV-induced repair mechanism also in S-phase cells.

To validate whether SPB10 indeed played a role in UV-related replication stress, we performed post-replication repair (PRR) comet assay under physiological conditions and following UV irradiation of non-targeting (siSCR) and *SPB10* siRNA-silenced U2OS cells and examined the presence of single-stranded DNA fragments generated by incomplete replication or UV light in S-phase cells following 0, 6, and 24 h of UV treatment. As expected, after UV irradiation, damaged DNA strands (the “tail” of the comet) were accumulated in non-targeting (siSCR) cells, but only a limited amount of DNA damage was detected in *SPB10* siRNA-silenced S-phase cells. Additionally, we observed faster repair, indicated by significantly shorter comet tails in the *SPB10* siRNA-silenced cells (Figure 3C,D). According to these results, our assumption is that in replicating cells, SPB10 is presumably involved in delaying the DNA repair process, thus facilitating a more precise repair mechanism to eliminate DNA damage.

### 2.4. SPB10 Is Associated with Chromatin

**Figure 4:**
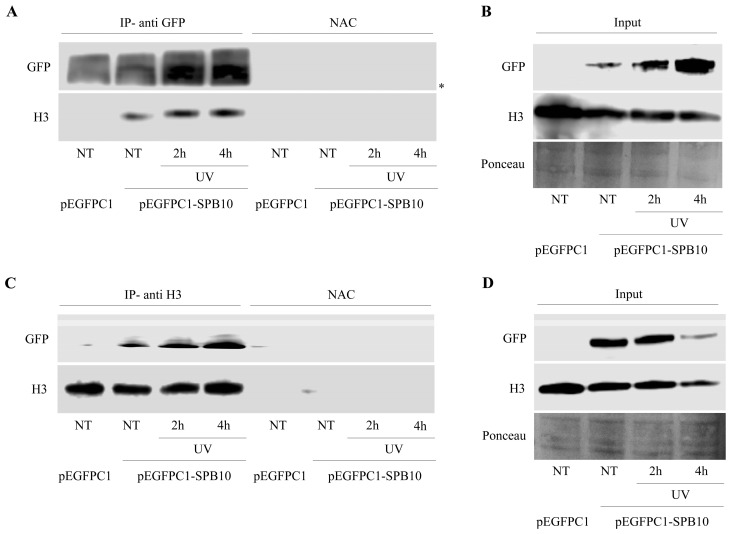
SPB10 shows interaction with H3: Immunoblot detection to reveal interaction between SPB10 and H3 in U2OS cells. Efficiency of immunoprecipitation experiment was controlled with (**A**) anti-GFP antibody (since cells were transfected with plasmid encoding SPB10-GFP fusion protein) and (**C**) anti-H3 antibody in precipitated samples in both control and UV-treated (2 and 4 h) samples. Asterisk shows unspecific bands. Western blots were performed on input protein samples to verify equal protein amount of each sample used for (**B**) SPB10-GFP and (**D**) H3 immunoprecipitation. Ponceau staining was used to detect equal loading.

The above-described data suggest that SPB10 might have an effect in the early steps of the UV-induced repair processes. To investigate whether this function takes place on the chromatin, we tested a possible association of SPB10 with the chromatin-related histone H3 protein following UV irradiation by co-IP in U2OS cells. For this, we transiently expressed the SPB10-GFP fusion protein in untreated and UV-irradiated (2 and 4 h) U2OS cells and performed an IP experiment using GFP antibody. Immunoprecipitated samples were analyzed by Western blot in order to detect the co-immunoprecipitated H3 protein. We found that regardless of the cellular conditions, SPB10 showed interaction with H3 histones (Figure 4A and Figure S2A). A verifying reciprocal co-IP experiment using a H3 specific antibody confirmed the interaction between H3 and SPB10 (Figure 4C and Figure S2B).

## 3. Discussion

In this study, we investigated a novel, yet-to-be-characterized function of the SPB10 protein following UV irradiation. Our preliminary microarray data indicated that members of the SerpinB family could play a role in UV-induced cellular responses [19,20]. We revealed an increase in the mRNA level of *SPB10* upon UV irradiation in three skin-derived cell lines (Hker E6SFM, HaCaT, and A375). Additionally, we demonstrated that the UV-triggered enhanced expression of *SPB10* is a general cellular response since we also observed this phenomenon in the U2OS osteosarcoma cell line, which is physiologically not exposed to UV light. This might indicate that the overexpression of *SPB10* upon UV irradiation is a common mechanism among different cell types possessing tissue-specific repair mechanisms [35,36]. In addition, we investigated the role of SPB10 in cell survival upon UV irradiation. We did not find a significant difference in the survival rate between non-targeting (siSCR) and *SPB10* siRNA-silenced U2OS cells. Although Przygodzka et al. found that under optimal growth conditions SPB10 has survival-promoting activity, this phenomenon is specific to myeloid cells, since ectopic expression of this Serpin in HT1080 fibroblasts did not change the proliferation rate of cells [31].

Although the *SPB10* mRNA level increased upon UV irradiation, suggesting its role in UV-induced cellular response, it does not influence cell viability. To examine this hypothesis, we studied the effect of SPB10 in UV-induced DNA damage repair in interphase and S-phase cells. We observed accelerated Nucleotide Excision Repair (NER) kinetics in interphase *SPB10* siRNA-silenced U2OS cells by detecting the XPC foci formation upon UV irradiation. By using post-replication repair (PRR) comet assay, we found accelerated UV-induced DNA repair in *SPB10* siRNA-silenced replicating cells. We also found that SPB10 influences the PCNA exchange on the chromatin, therefore presumably affecting the loading of the translesion polymerases. All these results suggest that SPB10 may have a function in the fine-tuned regulation of UV-triggered DNA repair. We suspect that SPB10 is involved in slowing down DNA repair, thus facilitating a more precise but prolonged repair mechanism both in interphase and in S-phase cells. Our assumption may also be supported by another example of the role of a serine protease in fine-tuning of DNA replication. Kirillova et al. demonstrated that TNF induces DNA replication in growth-arrested cells through NFκB, but this activation could be inhibited by N-tosyl-L-phenylalanine chloromethyl ketone (TPCK) serine protease inhibitor [37]. In addition, SPB10 is known to be an inhibitor of TNFα-induced cell death [38]. Furthermore, Kojima et al. reported that the serine protease FAM111A plays an important role in the removal of protein obstacles from DNA, thereby supporting the replicating fork progression. The knockout, or mutation, of *FAM111A* promotes replication fork stalling, leading to DNA damage accumulation, cell-cycle arrest in the G2/M phase, and eventual cell death [39]. 

Our assumption is that SPB10, as a serine protease inhibitor, may also take part in the regulation of FAM111A-mediated protein obstacle removal from the replication fork. Nevertheless, all of these data presume that SPB10 plays a role in UV-induced DNA damage repair, which is also supported by our finding of an interaction between SPB10 and the H3 core histone. Based on our data showing that the loss of SPB10 results in faster DNA repair, our assumption is that SPB10 could delay chromatin decondensation by interacting with H3. These results are in accordance with several studies demonstrating that regulatory proteins taking part in the early steps of the repair process exert their activity on the chromatin structure by binding to the nucleosomes. Among these proteins, 53BP1, RNF169, RAP80, and RAD18 were shown to bind to the ubiquitylated H2A histone, thereby participating in the DNA damage response and in the pathway choice between Non-homologous end joining and Homologous Recombination [40,41,42,43,44].

In this study, we analyzed the yet-uncharacterized function of the SPB10 protein in cellular UV response. Our findings suggested that SPB10 is a possible mediator involved in the UV-dependent modulation of the DNA repair rate.

## 4. Materials and Methods

### 4.1. Cell Lines, Media and Culturing Conditions

U2OS osteosarcoma, HaCaT keratinocyte, and A375 melanoma cells were cultured at 37 °C in Dulbecco’s Modified Eagle Medium (DMEM; Lonza, Basel, Switzerland) supplemented with 10% fetal bovine serum (Lonza, Basel, Switzerland), 4 mM L-glutamine (Sigma-Aldrich, St. Louis, MO, USA), and 1% antibiotic-antimycotic solution (Sigma-Aldrich, St. Louis, MO, USA) in a humidified atmosphere with 5% CO_2_.

Hker E6SFM keratinocyte cells were cultured at 37 °C in Keratinocyte SFM medium supplemented with L-glutamine, EGF, and BPE (Thermo Fisher Scientific, Waltham, MA, USA), 2 mM L-glutamine (Sigma-Aldrich, St. Louis, MO, USA), and 1% antibiotic-antimycotic solution (Sigma-Aldrich, St. Louis, MO, USA) in a humidified atmosphere with 5% CO_2_.

U2OS, HaCaT, and A375 cell lines were purchased from ATCC (Manassas, VA, USA), and Hker E6SFM cells were provided by Vilmos Tubak and were generated as described elsewhere in accordance with the relevant guidelines [21,22]. 

### 4.2. UV Irradiation

Cells were irradiated with Vilber Lourmat VL-/6.LM filtered UV lamps (Vilber Lourmat, Marne-la-Vallée, France). UV dosage was determined by a UVX Digital Ultraviolet Intensity Meter (Cole-Parmer, Vernon Hills, IL, USA) before each treatment. In this study, cells were irradiated with 16 mJ/cm^2^ (U2OS) and 80 mJ/cm^2^ UV (Hker E6SFM, A375, and HaCaT). The plate lids were removed during irradiation, then cells were washed with 1× PBS. After the treatment, 1× PBS was replaced with supplemented media and cells were incubated for 2, 6, 8, or 24 h, accordingly.

### 4.3. Microarray Experiment

The conditions used in the microarray experiment and the analysis methods are described in our recent paper (Ujfaludi, 2018) [19].

### 4.4. RNA Extraction, Reverse Transcription, and qRT-PCR

Total RNA samples were isolated with a ReliaPrep RNA Cell Miniprep System Kit (Promega, Madison, WI, USA) according to the manufacturer’s instructions. For each sample, 1 µg RNA was transcribed to cDNA by using TaqMan Reverse Transcription Reagent (Thermo Fisher Scientific, Waltham, MA, USA). Quantitative real-time PCR reactions were performed in a Thermo Scientific PicoReal Real-Time PCR System using SYBR Green chemistry (Thermo Fisher Scientific, Waltham, MA, USA). *SPB10*-specific primers 5′-CAAGCAAACCAGTGCAAATG-3′ and 5′-TAGGTGATGGCCTTTTCCAG-3′ were designed by using Primer3 software (Whitehead Institute for Biomedical Research), and 18S RNA primers were used as internal control [19]. The Ct values of samples were normalized to the internal control and alterations in mRNA levels were calculated by the ∆∆Ct method [45]. Data were obtained from three independent experiments.

### 4.5. Western Blot

Cell were harvested in lysis buffer (150 mM NaCl, 1% Triton X-100, 50 mM Tris-HCl, pH 8.0, and 1× PIC; Sigma-Aldrich, St. Louis, MO, USA) and incubated on ice for 1 h, followed by centrifugation (6000 rpm at 4 °C for 10 min). The supernatant lysate was supplemented with 6× SDS loading buffer containing 5% β-mercaptoethanol (Sigma-Aldrich, St. Louis, MO, USA) and boiled for 10 min. The samples were separated by SDS-PAGE and transferred to Amersham Hybond ECL-nitrocellulose membrane (GE Healthcare, Chicago, IL, USA). The following first antibodies were used: anti-H3 (Abcam, ab1791, Cambridge, UK) in 1:3000 dilution and anti-GFP (Abcam, ab6556, Cambridge, UK) in 1:1000 dilution. For chemiluminescent detection, secondary antibodies were applied: RAM-HRP (Dako, P0260, Santa Clara, CA, USA) and GAR-HRP (Dako, P0448, Santa Clara, CA, USA), followed by incubation with Immobilon Western Chemiluminescent HRP substrate (Merck Millipore, Burlington, MA, USA) and scanning using the Li-Cor 3600 C-DiGit Blot Scanner platform (Li-Cor, Lincoln, NE, USA). 

### 4.6. Cytoskeletal (CSK) Immunocytochemistry

Cells were washed with 1× PBS then incubated with CSK buffer 3 times for 3 min (10 mM Hepes pH 7.0, 100 mM sucrose, 3 mM MgCl_2_, 0.7% Triton X-100 (Sigma-Aldrich, St. Louis, MO, USA), and 0.3 mg/mL RNase A (Roche, Basel, Switzerland)) to eliminate all the non-chromatin-bound protein fractions. Cells were washed 3 times with 1× PBS, then fixed with 4% formaldehyde (Sigma-Aldrich, St. Louis, MO, USA) for 10 minutes. Then, the cells were permeabilized with 0.2% Triton X-100/PBS for 5 min. After washing steps, cells were blocked with 5% BSA (Sigma-Aldrich, St. Louis, MO, USA) in PBST (0.1% Tween 20 (Sigma-Aldrich, St. Louis, MO, USA) in PBS), supplemented with GAR-HPR (Dako, P0448, Santa Clara, CA, USA) and RAM-HPR (Dako, P0260, Santa Clara, CA, USA) in 1:2000 dilution for 20 min. Cells were washed with PBST, then incubated with primary antibodies diluted in 1% BSA/PBST: anti-PCNA (Santa Cruz, sc-7907, Santa Cruz, CA, USA) in 1:200 dilution, anti-RNAPII CTD 4H8 (Santa Cruz, sc-47701, Santa Cruz, CA, USA) in 1:100 dilution, and anti-XPC (Bethyl Laboratories, A301–122A, Montgomery, TX, USA) in 1:1000 dilution, which was kindly provided by Frederic Coin. After washing steps, fluorophore labeled secondary antibodies were used, like GAR Alexa 550 (Thermo Fisher Scientific, A21429, Waltham, MA, USA) and GAM Alexa 488 (Thermo Fisher Scientific, A11029, Waltham, MA, USA). After several washing steps with PBST, cells were mounted with DAPI-containing ProLong Gold antifade reagent (Thermo Fisher Scientific, Waltham, MA, USA). Samples were visualized with Olympus FluoView FV1000 confocal microscopy (Olympus Corporation, Tokyo, Japan). In case of image capturing, the same exposition-time was used for each sample. The captured images were quantified with ImageJ software (National Institutes of Health and the Laboratory for Optical and Computational Instrumentation, LOCI, University of Wisconsin, Madison, WI, USA).

### 4.7. Transfection

U2OS cells were seeded on plates 100 mm in diameter and transfected with 7.5 µg plasmid DNA using jetPEI transfection reagent (Polyplus, Illkirsch-Graffenstaden, France) according to the manufacturer’s instructions. Cells were incubated for 24 h at 37 °C and irradiated with UV light as described above. The applied plasmids were pEGFPC1 empty vector and pEGFPC1-SPB10 vector.

pEGFPC1-SPB10 vector was created using cloning primers containing the *SPB10* gene: forward 5′-GCGAGATCTGACTCTCTAGCAACATCA-3′ and reverse 5′-ATGTCGACTTAGGGGGAGCATAATCT-3′ sequences, supplemented with BglII and SalI restriction endonuclease sites, respectively.

We amplified the SPB10 protein coding gene region with the above described primers using PCR (98 °C for 10 s, 65 °C for 30 s, 72 °C for 1 min, for 40 cycles). The PCR products were ligated into cloning vector using CloneJET PCR Cloning Kit (Thermo Fisher Scientific, Waltham, MA, USA) according to the manufacturer’s instructions. The *SPB10* insert was incorporated into the pEGFP-C1 vector using BglII and SalI endonucleases.

### 4.8. Co-Immunoprecipitation

Cells were lysed in lysis buffer (150 mM NaCl, 1% Triton X-100, 50 mM Tris-HCl, pH 8.0; Sigma-Aldrich, St. Louis, MO, USA) supplemented with 1× PIC (Sigma-Aldrich, St. Louis, MO, USA) and 1× PhosSTOP (Roche, Basel, Switzerland) and incubated on ice for 1 h, then centrifuged (6000 rpm at 4 °C for 5 min). Afterwards, 300 μg of the supernatant lysates were pre-cleared for 2 h with blocked Protein A-Sepharose beads (Sigma-Aldrich, St. Louis, MO, USA). For immunoprecipitation, anti GFP-antibody (Abcam, ab6556, Cambridge, UK) or anti H3-antibody (Abcam, ab1791, Cambridge, UK) was used. IgG control was used for detection of nonspecific protein binding. For this, Protein A-Sepharose beads were added to the cell lysate without a specific antibody. The protein-antibody complexes were captured with 40 μL of Protein A-Sepharose beads. In the following steps, beads were washed 4 times with lysis buffer supplemented with 1× PIC (Sigma-Aldrich, St. Louis, MO, USA) and 1× PhosSTOP (Roche, Basel, Switzerland). After washing steps, protein-protein complexes were eluted from the beads by boiling the samples in 6× SDS loading buffer containing 5% β-mercaptoethanol (Sigma-Aldrich, St. Louis, MO, USA) for 10 min. The beads were removed by centrifugation (13,000 rpm at 4 °C for 5 min) and the supernatants were run on PAGE.

### 4.9. Viability Assay

U2OS cells were collected, then resuspended in fetal bovine serum (Lonza, Basel, Switzerland) and centrifuged (2000 rpm at room temperature for 10 min). The collected cells were stained with a 1:1 proportion of 0.4% Trypan Blue dye (Sigma-Aldrich, St. Louis, MO, USA) and DMEM (Lonza, Basel, Switzerland) staining solution and incubated for 15 min. The ratio of live and dead cells was counted in a Burker chamber. 

### 4.10. siRNA Transfection

U2OS cells were seeded into 6-well plates and the following day, when the confluence reached 30–50%, the cells were transfected with siRNA using INTERFERin transfection reagent (Polyplus, Illkirsch-Graffenstaden, France) according to the manufacturer’s instructions. For each well, the siRNA and INTERFERin were diluted into 200–200 μL of serum-free DMEM. The two solutions were mixed and incubated for 20 min at 25 °C. Meanwhile, the growth medium on the cells was replaced with antibiotic-free supplemented DMEM. Then, the siRNA and INTERFERin-containing solution was added to the cells and homogenized gently. Cells were incubated for 48 h at 37 °C and were irradiated with UV light as described above. The applied siRNAs were 100 nM of *SPB10* siRNA pool (L-019923-02-0005, Dharmacon, Lafayette, CO, USA) for *SPB10* gene silencing, and 25 nM of non-targeting scramble (SCR) siRNA pool (D-001810-10-05, Dharmacon, Lafayette, CO, USA) for negative control.

### 4.11. Post-Replication Repair (PRR) Comet Assay

U2OS cells were seeded into 6-well plates and transfected with siRNA as described above. After 72 h of incubation, the growth medium was replaced with fresh medium containing 20 μM bromodeoxyuridine (BrdU) for 20 min at 37 °C to visualize replication forks in the S-phase cells. Then, the cells were washed two times with 1× PBS and irradiated with UV light. After the treatment, cells were chased in fresh medium containing 200 μM of 4× dNTPs and incubated for 0, 6 (also basal condition (NT) samples), and 24 h at 37 °C, then cells were sedimented (200× *g* at 25 °C for 5 min). The collected cells were resuspended in 0.75% low-melting agarose (Sigma-Aldrich, St. Louis, MO, USA) and maintained at 37 °C. Afterward, the cell suspension was spread on agarose-precoated and dried slides and incubated for 3 min at 4 °C to solidify. The slides were then incubated in an ice-cold lysis solution (2.5 M NaCl, 0.1 M EDTA, 10 mM Tris-HCl, pH 10.0, 1% Triton X-100 and 0.5% N-lauroylsarcosine sodium salt (Sigma-Aldrich, St. Louis, MO, USA)) for 1.5 h at 4 °C in a Coplin jar to get rid of the cell membranes and proteins, followed by 3 washing steps with 400 mM Tris-HCl buffer at pH 7.4 (Sigma-Aldrich, St. Louis, MO, USA) to remove the remaining cell debris. Subsequently, the slides were incubated in electrophoretic buffer (300 mM NaOH, 1 mM EDTA, pH 13 (Sigma-Aldrich, St. Louis, MO, USA)) for 40 min, then electrophoresis was performed at 1 V/cm (25 V, 300 mA) for 20 min. The samples were blocked with 1% BSA in PBST (0.1% Tween 20 (Molar Chemicals, Halásztelek, Hungary) in PBS) for 15 min, then incubated with anti-BrdU (OBT0030G, Bio-Rad Laboratories, Berkeley, CA, USA) primary antibody at 1:300 dilution for 1.5 h. After washing steps, GAR Alexa 488 (A-11006, Thermo Fisher, Waltham, MA, USA) secondary antibody at 1:400 dilution was used. Both primary and secondary antibodies were diluted in 1% BSA-PBST. Samples were visualized with a Zeiss Axioscope fluorescent microscope (Zeiss, Jena, Germany). The same exposure time was used to capture each image. Komet 5.0 image analysis software (Kinetic Imaging Ltd., Liverpool, UK) was used to analyze DNA fragmentation. Data were derived from 100 cells in each condition.

## Supplementary Materials

The following are available online at https://www.mdpi.com/article/10.3390/ijms22168500/s1.

## Abbreviations

6-4 PP	6-4 pirimidine-pirimidon photoproduct
BrdU	bromodeoxyuridine
CPD	cyclobutane-pyrimidine dimers
co-IP	co-immunoprecipitation
DMEM	Dulbecco’s Modified Eagle Medium
GFP	green fluorescent protein
NER	Nucleotide Excision Repair
NT	non-treated (basal condition)
PCNA	proliferating cell nuclear antigen
PRR comet assay	post-replication repair comet assay
qPCR	quantitative polymerase chain reaction
siSCR	non-targeting scrambled siRNA
SPB	SerpinB
SPB2	SerpinB2
SPB10	SerpinB10 (Bomapin)
SPB13	SerpinB13
TLS DNA Polymerase	translesion DNA polymerase
TPCK	N-tosyl-L-phenylalanine chloromethyl ketone
XPC	Xeroderma Pigmentosum C

## References

[1] Epstein J.H. (1983). Photocarcinogenesis, skin cancer, and aging. J. Am. Acad. Dermatol..

[2] de Gruijl F.R., van Kranen H.J., Mullenders L.H. (2001). UV-induced DNA damage, repair, mutations and oncogenic pathways in skin cancer. J. Photochem. Photobiol. B.

[3] Kielbassa C., Roza L., Epe B. (1997). Wavelength dependence of oxidative DNA damage induced by UV and visible light. Carcinogenesis.

[4] Rünger T.M., Kappes U.P. (2008). Mechanisms of mutation formation with long-wave ultraviolet light (UVA). Photodermatol. Photoimmunol. Photomed..

[5] de Gruijl F.R. (2000). Photocarcinogenesis: UVA vs UVB. Methods Enzymol..

[6] Kabuyama Y., Homma M.K., Kurosaki T., Homma Y. (2002). Early signaling events induced by 280-nm UV irradiation. Eur. J. Biochem..

[7] Pustovalova Y., MacIejewski M.W., Korzhnev D.M. (2013). NMR mapping of PCNA interaction with translesion synthesis DNA polymerase Rev1 mediated by Rev1-BRCT domain. J. Mol. Biol..

[8] Waters L.S., Minesinger B.K., Wiltrout M.E., D’Souza S., Woodruff R.V., Walker G.C. (2009). Eukaryotic Translesion Polymerases and Their Roles and Regulation in DNA Damage Tolerance. Microbiol. Mol. Biol. Rev..

[9] Guo C., Kosarek-Stancel J.N., Tang T.S., Friedberg E.C. (2009). Y-family DNA polymerases in mammalian cells. Cell. Mol. Life Sci..

[10] Hoege C., Pfander B., Moldovan G.-L., Pyrowolakis G., Jentsch S. (2002). RAD6-dependent DNA repair is linked to modification of PCNA by ubiquitin and SUMO. Nature.

[11] Moldovan G.L., Pfander B., Jentsch S. (2007). PCNA, the Maestro of the Replication Fork. Cell.

[12] Rizzo A.A., Korzhnev D.M. (2019). The Rev1-Polζ translesion synthesis mutasome: Structure, interactions and inhibition. Enzymes.

[13] Acharya N., Patel S.K., Sahu S.R., Kumari P. (2020). “PIPs” in DNA polymerase: PCNA interaction affairs. Biochem. Soc. Trans..

[14] Friedberg E.C., Lehmann A.R., Fuchs R.P.P. (2005). Trading Places: How do DNA polymerases switch during translesion DNA synthesis?. Mol. Cell.

[15] Zhuang Z., Johnson R.E., Haracska L., Prakash L., Prakash S., Benkovic S.J. (2008). Regulation of polymerase exchange between Polη and Polδ by monoubiquitination of PCNA and the movement of DNA polymerase holoenzyme. Proc. Natl. Acad. Sci. USA.

[16] Maiorano D., El Etri J., Franchet C., Hoffmann J.-S. (2021). Translesion Synthesis or Repair by Specialized DNA Polymerases Limits Excessive Genomic Instability upon Replication Stress. Int. J. Mol. Sci..

[17] Guilliam T.A., Yeeles J.T.P. (2020). Reconstitution of translesion synthesis reveals a mechanism of eukaryotic DNA replication restart. Nat. Struct. Mol. Biol..

[18] Cortez D. (2019). Replication-Coupled DNA Repair. Mol. Cell.

[19] Ujfaludi Z., Tuzesi A., Majoros H., Rothler B., Pankotai T., Boros I.M. (2018). Coordinated activation of a cluster of MMP genes in response to UVB radiation. Sci. Rep..

[20] Majoros H., Ujfaludi Z., Borsos B.N., Hudacsek V.V., Nagy Z., Coin F., Buzas K., Kovács I., Bíró T., Boros I.M. (2019). SerpinB2 is involved in cellular response upon UV irradiation. Sci. Rep..

[21] Polyanka H., Szabo K., Tax G., Tubak V., Kusz E., Ujfaludi Z., Boros I., Bata-Csorgo Z., Kemény L., Szell M. (2018). Primary characterization of a novel HPV-E6 oncogene immortalized keratinocyte cell line. J. Investig. Dermatol..

[22] Szlavicz E., Szabo K., Groma G., Bata-Csorgo Z., Pagani F., Kemeny L., Szell M. (2017). Splicing factors differentially expressed in psoriasis alter mRNA maturation of disease-associated EDA+ fibronectin. Mol. Cell. Biochem..

[23] Silverman G.A., Whisstock J.C., Askew D.J., Pak S.C., Luke C.J., Cataltepe S., Irving J.A., Bird P.I. (2004). Human clade B serpins (ov-serpins) belong to a cohort of evolutionarily dispersed intracellular proteinase inhibitor clades that protect cells from promiscuous proteolysis. Cell. Mol. Life Sci..

[24] Baker M.S., Bleakley P., Woodrow G.C., Doe W.F. (1990). Inhibition of cancer cell urokinase plasminogen activator by its specific inhibitor PAI-2 and subsequent effects on extracellular matrix degradation. Cancer Res..

[25] Sheng S., Truong B., Fredrickson D., Wu R., Pardee A.B., Sager R. (1998). Tissue-type plasminogen activator is a target of the tumor suppressor gene maspin. Proc. Natl. Acad. Sci. USA.

[26] Zhang P., Li X., He Q., Zhang L., Song K., Yang X., He Q., Wang Y., Hong X., Ma J. (2020). TRIM21-SERPINB5 AIDS GMPS repression to protect nasopharyngeal carcinoma cells from radiation-induced apoptosis. J. Biomed. Sci..

[27] Mo Y., Zhang K., Feng Y., Yi L., Liang Y., Wu W., Zhao J., Zhang Z., Xu Y., Hu Q. (2019). Epithelial serpinb10, a novel marker of airway eosinophilia in asthma, contributes to allergic airway inflammation. Am. J. Physiol.-Lung Cell. Mol. Physiol..

[28] Ahn J.W., Atwell B.J., Roberts T.H. (2009). Serpin genes AtSRP2 and AtSRP3 are required for normal growth sensitivity to a DNA alkylating agent in Arabidopsis. BMC Plant Biol..

[29] Hsieh H.-H., Chen Y.-C., Jhan J.-R., Lin J.-J. (2017). The serine protease inhibitor serpinB2 binds and stabilizes p21 in senescent cells. J. Cell Sci..

[30] Jiang J., Ding Y., Wu M., Chen Y., Lyu X., Lu J., Wang H., Teng L. (2020). Integrated genomic analysis identifies a genetic mutation model predicting response to immune checkpoint inhibitors in melanoma. Cancer Med..

[31] Przygodzka P., Ramstedt B., Tengel T., Larsson G., Wilczynska M. (2010). Bomapin is a redox-sensitive nuclear serpin that affects responsiveness of myeloid progenitor cells to growth environment. BMC Cell Biol..

[32] Chou R.-H., Wen H.-C., Liang W.-G., Lin S.-C., Yuan H.-W., Wu C.-W., Chang W.-S.W. (2011). Suppression of the invasion and migration of cancer cells by SERPINB family genes and their derived peptides. Oncol. Rep..

[33] Shioji G., Ezura Y., Nakajima T., Ohgaki K., Fujiwara H., Kubota Y., Ichikawa T., Inoue K., Shuin T., Habuchi T. (2005). Nucleotide variations in genes encoding plasminogen activator inhibitor-2 and serine proteinase inhibitor B10 associated with prostate cancer. J. Hum. Genet..

[34] Sale J.E., Lehmann A.R., Woodgate R. (2012). Y-family DNA polymerases and their role in tolerance of cellular DNA damage. Nat. Rev. Mol. Cell Biol..

[35] Garmyn M., Young A.R., Miller S.A. (2018). Mechanisms of and variables affecting UVR photoadaptation in human skin. Photochem. Photobiol. Sci..

[36] López-Camarillo C., Aréchaga Ocampo E., López Casamichana M., Pérez-Plasencia C., Álvarez-Sánchez E., Marchat L.A. (2011). Protein Kinases and Transcription Factors Activation in Response to UV-Radiation of Skin: Implications for Carcinogenesis. Int. J. Mol. Sci..

[37] Kirillova I., Chaisson M., Fausto N. (1999). Tumor necrosis factor induces DNA replication in hepatic cells through nuclear factor κB activation. Cell Growth Differ..

[38] Schleef R.R., Chuang T.L. (2000). Protease Inhibitor 10 Inhibits Tumor Necrosis Factor α-induced Cell Death. J. Biol. Chem..

[39] Kojima Y., Machida Y., Palani S., Caulfield T.R., Radisky E.S., Kaufmann S.H., Machida Y.J. (2020). FAM111A protects replication forks from protein obstacles via its trypsin-like domain. Nat. Commun..

[40] Borsos B.N., Majoros H., Pankotai T. (2020). Ubiquitylation-Mediated Fine-Tuning of DNA Double-Strand Break Repair. Cancers.

[41] Doil C., Mailand N., Bekker-Jensen S., Menard P., Larsen D.H., Pepperkok R., Ellenberg J., Panier S., Durocher D., Bartek J. (2009). RNF168 Binds and Amplifies Ubiquitin Conjugates on Damaged Chromosomes to Allow Accumulation of Repair Proteins. Cell.

[42] Stewart G.S., Panier S., Townsend K., Al-Hakim A.K., Kolas N.K., Miller E.S., Nakada S., Ylanko J., Olivarius S., Mendez M. (2009). The RIDDLE Syndrome Protein Mediates a Ubiquitin-Dependent Signaling Cascade at Sites of DNA Damage. Cell.

[43] Mattiroli F., Vissers J.H.A., Van Dijk W.J., Ikpa P., Citterio E., Vermeulen W., Marteijn J.A., Sixma T.K. (2012). RNF168 ubiquitinates K13-15 on H2A/H2AX to drive DNA damage signaling. Cell.

[44] Gatti M., Pinato S., Maspero E., Soffientini P., Polo S., Penengo L. (2012). A novel ubiquitin mark at the N-terminal tail of histone H2As targeted by RNF168 ubiquitin ligase. Cell Cycle.

[45] Johnson M.R., Wang K., Smith J.B., Heslin M.J., Diasio R.B. (2000). Quantitation of Dihydropyrimidine Dehydrogenase Expression by Real-Time Reverse Transcription Polymerase Chain Reaction. Anal. Biochem..

