# Supplementary material for: SerpinB10, a Serine Protease Inhibitor, Is Implicated in UV-Induced Cellular Response
Source: Int J Mol Sci. 2021 Aug 7;22(16):8500. doi: 10.3390/ijms22168500 (PMC8395218; doi:10.3390/ijms22168500)
Supplement: Supplementary file 1 [file ijms-22-08500-s001.zip › ijms-1201816-supplementary.pdf]

gene symbol	fold change ($\log_2$)	gene symbol	fold change ($\log_2$)
SerpinA1	no change	*SerpinB2*	1.495
SerpinA2	no change	*SerpinB5*	no change
SerpinA4	no change	*SerpinB9*	no change
SerpinA5	no change	*SerpinB10*	4.253
SerpinA6	no change	*SerpinB13*	1.180
SerpinA8	no change	*SerpinC1*	no change
SerpinA9	no change	*SerpinD1*	no change
SerpinA10	no change	*SerpinF2*	no change
SerpinA11	no change	*SerpinF3*	no change
SerpinA12	no change	*SerpinG1*	no change
SerpinA13	no change	*SerpinH1*	no change

Figure S1: UV irradiation contributes to overexpression of *SPB2*, *SPB10,* and *SPB13* genes (80 mJ/cm^2): UV damage-induced $\log_2$ expression changes of the Serpin family members in Hker E6SFM keratinocytes detected by microarray.

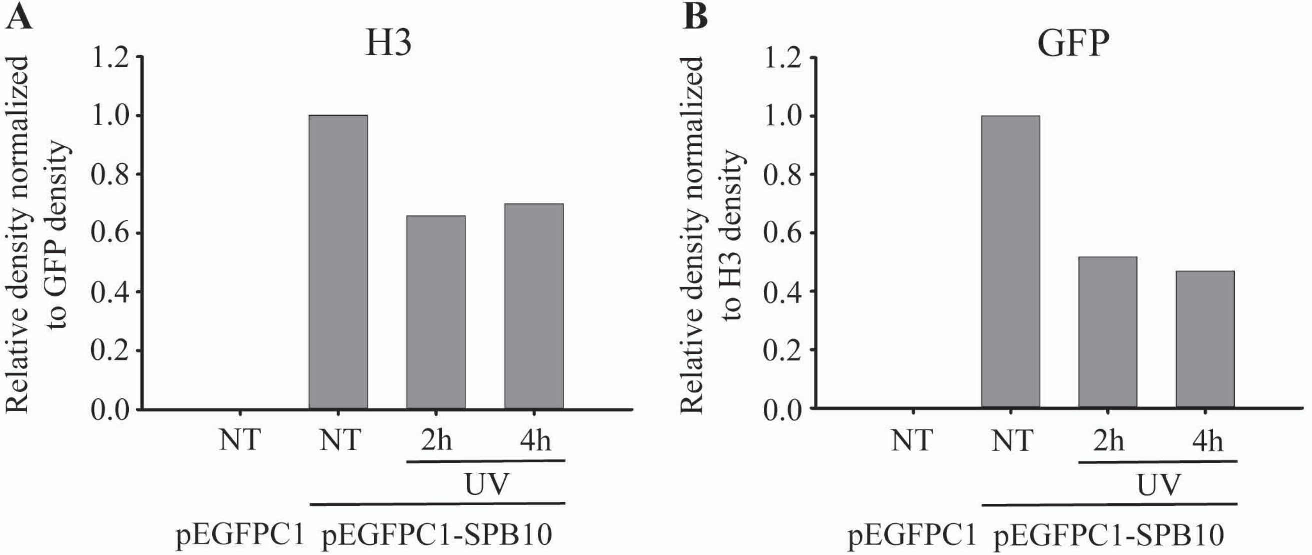

Figure S2: Relative density of chemiluminescent signals detected with (A) anti-H3 and (B) anti-GFP antibody in Western blots shown in Figure 4 was normalized to signal density of immunoprecipitated (A) GFP and (B) H3 with Fiji ImageJ.
